# Chemical Characterization of “*Alcaparras*” Stoned Table Olives from Northeast Portugal

**DOI:** 10.3390/molecules16119025

**Published:** 2011-10-26

**Authors:** Anabela Sousa, Susana Casal, Albino Bento, Ricardo Malheiro, M. Beatriz P.P. Oliveira, José Alberto Pereira

**Affiliations:** 1 Mountain Research Centre (CIMO), School of Agriculture, Polytechnic Institute of Bragança, Campus Stª Apolónia, Apartado 1172, Bragança 5301-855, Portugal; Email: anabela.sousa@ipb.pt (A.S.); bento@ipb.pt (A.B.); rmalheiro@ipb.pt (R.M.); 2 REQUI*M*TE/Laboratory of Bromatology and Hydrology, Faculty of Pharmacy, Porto University, Rua Aníbal Cunha 164, Porto 4050-047, Portugal; Email: beatoliv@ff.up.pt

**Keywords:** *Olea europaea *L., stoned table olives, fatty acids, tocopherols, phytochemicals

## Abstract

Commercial stoned table olives named “*alcaparras*” from Trás-os-Montes (Portugal) were chemically characterized. During three consecutive years (2004–2006) 30 samples (10 per year) were examined for their nutritional value (moisture, crude protein, total fat, ash, carbohydrates, and energy), with a detailed report of the fatty acids and tocopherols composition. Water was the major constituent (72.5 ± 5.5%), followed by fat (14.6 ± 5.1%). The average amount of protein and ash were 1.1% and 3.4%, respectively, reporting unusual ash values for table olives, related to the technological process. One hundred grams of fresh stoned table olives presented an average energetic value of 156 kcal, lower than most table olives. The lipids are rich in oleic acid (average of 77.7 ± 2.0%), followed by palmitic acid and linoleic acid. Samples showed an average of total tocopherols of 1.2 mg/100 g of fresh weight, being α-tocopherol the most abundant. Table olives are important sources of MUFA, as olive oil, recognized as a preventive factor in diseases in which free radicals are implicated, complemented by the amounts of vitamin E, with both antioxidant and vitamin action.

## 1. Introduction

In the last decades olive products, particularly olive oil, the finest olive product, and table olives, have been attracting increasing interest, due mostly to reports on their health promoting effects [1]. Table olives are important constituents of the Mediterranean diet, being processed by several classical and traditional methodologies. According to the Trade Standard Applying to Table Olives [2] table olives are the product “prepared from the sound fruits of varieties of the cultivated olive tree (*Olea europaea *L.) which are chosen for their production olives whose volume, shape, flesh-to-stone ratio, fine flesh, taste, firmness and ease of detachment from the stone make them particularly suitable for processing”. According to the ripeness stage of the fruit, trade preparation, styles and sizing, different kinds of table olives can and should be classified.

Besides the classical, green (Spanish) and black (Californian) processing methods, different kinds of traditional methods are also used in Portugal. In the Trás-os-Montes region (Northeast Portugal), the second largest national producing area, stoned halved olives known as “*alcaparras*” are largely produced by local growers, using domestic or small-scale facilities, commercialized in local markets, and latter consumed seasoned with herb spices, onion, garlic, vinegar and olive oil, mostly in the same winter season, due to their reduced-shelf life. This kind of table olives are processed from green or yellow-green healthy olive fruits, which are broken using a wood hammer, being the pulp and stone separated, and therefore classified under the “stoned halved olives” descriptor considered by the Trade Standard [2]. The pulp is sliced into two approximately equal parts, perpendicularly to the major axis of the fruit, and placed in water, which is changed three or four times during a week. The applied treatment removes olives bitterness; they are then preserved in brine.

Different works have studied table olives and the influence of some aspects such as preparation methods [3,4,5], ripening time [6,7], cultivar [7,8,9,10], and agronomical aspects [11] on their composition, 

Table olives are mainly constituted of water, followed by fat, carbohydrates, proteins and ash. From a nutritional point of view, table olives are well-known sources of compounds with beneficial relevance. These benefits are associated with their fatty acids content, mainly monounsaturated fatty acids, and to minor constituents such as tocopherols, phenolic compounds and phytosterols [12,13].

Particular attention has been given to polyphenolic composition [4,14,15,16], due to their bioactive antioxidant activity, but fewer studies are dedicated to table olive tocopherols, the main lipophilic antioxidant [3,7,17]. Many studies describe α-tocopherol as having a protective action on human health against different pathologies, contributing to minimize the adverse effects of inflammatory diseases by defending the body against free radicals [18,19].

In the last few years our research group began to characterize stoned table olives from the Trás-os-Montes region. This type of green processed olives revealed appreciable amounts of phenolics [20], with three flavonoidic compounds identified in aqueous extracts, namely, luteolin 7-*O*-glucoside, apigenin 7-*O*-glucoside, and luteolin. Aqueous extracts from stoned table olives also revealed antimicrobial activity against several microorganisms that may be causal agents of human intestinal and respiratory tract infections [21] and appreciable antioxidant capacity against free radicals [21,22]. Despite their richness in bioactive compounds, table olives are also regarded as a source of fat and salt, highly dependent on the olive cultivar and their ripening stage, for the former, as well as the processing method for the latter.

The “*alcaparras*” processing method presents some similarities with that used for the *Kalamata* type of olive [23], in the sense that olives are cut, debittered by contact with water and thereafter conserved in brine (8%) to avoid fermentation. Nevertheless, the latter process uses pink to purple olives, at a higher maturation stage, and therefore with higher fat content. Therefore, this traditional processing method finds no totally equivalents among published data.

The aim of the present work was to contribute for the chemical characterization of traditional “*alcaparras*” stoned table olives produced and commercialized in the Trás-os-Montes region. For this purpose, a total of 30 samples, produced and commercialized in three different years (2004, 2005 and 2006), were purchased at regional markets, and studied for nutritional value, individual fatty acids and tocopherol contents.

## 2. Results and Discussion

### 2.1. Proximate Chemical Composition

The results obtained in the proximate chemical composition (grams per 100 g of fresh weight) of commercial“*alcaparras*” stoned table olives from three different years of sampling are reported in Table 1.

Water was the major component of these traditional table olives, varying from 58.9% to 80.0%, with 72.5 ± 5.5% on average for the three years. Despite having the pulp in direct contact with the brine, “*alcaparras*” stoned table olives moisture was similar to other one-piece table olives [17,23]. This factor could be attributed to the partially solubilization of pectic substances imposed by the lye treatment on the drupe [23], resulting in cell rupture and reduced pulp consistency, a situation not observed in the studied table olives. Fat was the second most abundant component, ranging from 7.6% to 29.3%, with marked differences observed within and between the years in study (*P* < 0.05).

**Table 1:** Proximate chemical composition (grams per 100 g of fresh weight) of “*alcaparras*” stoned table olives samples from three different years of production.

Samples	Moisture	Total fat	Crude protein	Ash	Total sugars *
2004					
*1*	74.8 ± 0.5	13.4 ± 0.0	-	-	
*2*	74.5 ± 0.2	14.2 ± 0.0	-	-	
*3*	78.3 ± 0.5	14.3 ± 0.0	-	-	
*4*	71.6 ± 1.6	17.6 ± 0.0	-	-	
*5*	75.1 ± 0.7	13.8 ± 0.0	-	-	
*6*	66.8 ± 3.3	15.7 ± 0.0	-	-	
*7*	74.1 ± 1.6	13.1 ± 0.0	-	-	
*8*	70.1 ± 1.5	22.0 ± 0.0	-	-	
*9*	72.6 ± 1.4	9.1 ± 0.0	-	-	
*10*	73.7 ± 1.8	12.6 ± 0.0	-	-	
Average	**73.2 ± 3.2 a**	**14.6 ± 0.0 a,b**	**-**	**-**	
2005					
*11*	72.2 ± 0.2	13.3 ± 1.6	0.9 ± 0.0	5.6 ± 0.0	5.3
*12*	69.0 ± 2.8	21.4 ± 0.2	1.4 ± 0.0	1.8 ± 0.1	3.6
*13*	73.3 ± 0.9	12.9 ± 3.6	0.9 ± 0.0	3.6 ± 0.0	6.5
*14*	73.2 ± 1.3	10.8 ± 0.8	0.8 ± 0.0	3.1 ± 0.3	9.4
*15*	71.8 ± 0.6	10.4 ± 4.6	0.8 ± 0.0	4.7 ± 1.1	9.6
*16*	70.2 ± 0.9	18.5 ± 1.0	0.9 ± 0.0	3.6 ± 0.4	4.1
*17*	59.5 ± 0.1	28.4 ± 4.3	1.7 ± 0.0	1.8 ± 0.8	5.3
*18*	58.9 ± 0.5	29.3 ± 2.1	1.5 ± 0.0	5.1 ± 0.5	2.3
*19*	60.3 ± 1.6	22.8 ± 0.1	1.7 ± 0.0	2.9 ± 0.1	9.6
*20*	65.7 ± 0.7	18.7 ± 0.8	1.5 ± 0.0	1.7 ± 0.0	9.7
Average	**67.5 ± 5.9** ** b**	**18.3 ± 6.1 a**	**1.2 ± 0.4 a**	**3.5 ± 1.4 a**	**6.5 ± 2.8**
2006					
*21*	77.3 ± 0.5	10.4 ± 1.6	1.3 ± 0.0	2.8 ± 0.1	5.5
*22*	67.0 ± 0.1	17.8 ± 0.4	1.4 ± 0.0	2.3 ± 0.1	8.7
*23*	74.7 ± 0.2	15.8 ± 0.2	1.0 ± 0.0	1.0 ± 0.0	4.8
*24*	75.2 ± 0.9	14.2 ± 0.4	1.0 ± 0.0	1.5 ± 0.1	5.3
*25*	77.4 ± 0.8	9.5 ± 0.7	0.8 ± 0.0	8.3 ± 0.0	1.2
*26*	80.0 ± 0.4	7.6 ± 0.2	1.0 ± 0.0	3.3 ± 0.1	5.1
*27*	78.7 ± 0.3	9.4 ± 2.8	1.1 ± 0.0	4.6 ± 0.1	3.5
*28*	73.3 ± 0.7	17.6 ± 0.9	1.2 ± 0.0	1.2 ± 0.0	4.0
*29*	78.7 ± 0.9	9.1 ± 0.2	1.0 ± 0.0	4.6 ± 0.1	3.8
*30*	76.6 ± 0.1	14.0 ± 0.2	0.9 ± 0.0	2.1 ± 0.0	3.6
Average	**76.0 ± 3.5 a**	**12.6 ± 3.8 b**	**1.1 ± 0.2 a**	**3.2 ± 2.2 a**	**4.6 ± 1.9**

Different letters in the same row indicates significant differences between years (*P* < 0.05); * Estimated.

With an average of 15.3 ± 5.4% fresh weight, or 54.9 ± 11.5% when expressed on a dry base, these figures are in accordance with literature [17,23,33]. The higher variability however, could be explained by the diversity of the samples acquired, with different maturation stages, olive cultivars, and different storage periods, among other important factors. Moreover, commercial “*alcaparras*” stoned table olives are blends of several olive cultivars in unknown relative proportions. The higher lipid amounts in the 2005 samples, particularly samples 17 and 18, could be attributed to the presence of higher amounts of a specific olive cultivar, such as Cordovil, also occuring in the region. The lower values however, from 8 to 12%, are slightly below those expected for processed table olives. The information on total lipids provided by López-López *et al*. [34] for diversified commercial table olives, included also values up to 8.7%, but these were from stuffed olives, therefore not comparable. An important issue is the olive maturation state, with the fat content increasing during olive maturation process [35]. Being prepared with green olives, their fat content should be reduced. According to Ünal and Nergiz [23] the fat amount usually remains constant during processing and storage of one-piece table olives. Once “*alcaparras*” stoned table olives are processed with the pulp broken in pieces and in direct contact with water, a reduction in the fat amount could occur. 

“*Alcaparras*” stoned table olives are typically produced using several cultivars, no attention being paid to the adequacy of the different cultivars for this processing treatment, using both suitable olives with higher pulp firmness such as *Cv*. Cobrançosa, and Santulhana or *Cv*. Negrinha de Freixo, typical in the region, with others less adequate for the purpose, such as *Cv*. Verdeal Transmontana or even *Cv*. Madural. This parameter was not evaluated in our study because, as explained, the samples were mixtures of different olives cultivars, acquired after processing, as available to consumers.

Crude protein average values were comparable in the samples analysed (n = 20), with a mean of 1.1 ± 0.3%, in accordance with the values reported for other table olives [17,23]. The high variability, particularly due to the increased amounts in samples 17 to 20, is attributed to their lower moisture content, influencing all the parameters expressed on a fresh weight basis. When expressed on a dry basis these samples presented equivalent amounts of protein. Protein was not evaluated in the first sampling year. Indeed, the initial objective was to address the variability in the fat constituents, the major nutritional component, but the results for the first year highlighted the possibility of a higher variability than expected for the nutritional composition, particularly regarding protein and added salt, so all these parameters were evaluated in the subsequent sampling years but the 2004 samples were not adequately preserved, hindering the possibility to complete with confidence the analyses. 

Ash content was the parameter presenting the highest variability, with values ranging from 1.0% to 8.3%, regardless of the equivalent average between the two sampling years, with 3.4% in 2005 and 3.2% in 2006. After processing, and for longer durability and safety for commercial purposes, “*alcaparras” *stoned table olives are preserved in brine, with NaCl additions based on the producer’s experience and perception. Therefore, and depending on the brine concentration and storage time, the amount of NaCl in the pulp is expected to increase, based on osmotic diffusion. Despite the absence of specific data regarding diffusion from brine into olives, particularly in cracked olives, the data of Ünal and Nergiz [23], for *Kalamata* type olives, suggests that the amounts of NaCl are comparatively lower than those reported for green and black type processed olives, for all the storage times evaluated. Indeed, the NaCl amounts reported immediately after processing for those olive types, around 4%, were only achieved in *Kalamata* type after a storage period of 16 months. Our mean results are comparable to the *Kalamata* type, and slightly lower than those reported for other one-piece processed olives [17,23]. Although an important factor for microbial safety, high salt values are nutritionally undesirable, in light of current concerns about sodium intake due to its association with hypertension and cancer, among other important diseases in our society. The brine standardization to the safely smaller concentrations is an important issue to address in future studies regarding these traditional table olives.

Total sugars and crude fiber are present in low amounts in table olives, as addressed by many authors, and particularly by López-López *et al*. [36] in a detailed study on Spanish commercial samples. Fiber is usually within narrow values, with average amounts of 2.75% for the diversified green table olives in the previous study. Therefore, this value was used to estimate the total sugar amount in our samples by difference to 100% fresh weight, after deducting moisture, fat, protein and ash. The estimated total sugars are detailed in Table 1 where, despite the variability observed between samples, the mean contents are similar, with 6.5% in 2005 and 4.6% in 2006. Such results are in accordance to those referred by Kailis and Harris [37] (from 8 and 12% for different raw olives). When compared to other processing methods like Spanish-style green olives in brine [23] and Greek-style naturally black olives in brine [38], “*alcaparras*” stoned table olives presented higher contents of carbohydrates. Once more, a technological factor can explain the differences observed. The mentioned styles, two of the most common table olives preparations found and commercialized worldwide, involve fermentative processes, with a consequent reduction in the sugar content.

Based on the fat, protein, estimated carbohydrates and fiber amounts, an energy content of 156 kcal was per 100 g of “*alcaparras*” stoned table olives was calculated. Nevertheless, high variations are reported regarding energetic value, ranging from 91 kcal (Sample 16) to 283 kcal (Sample 7), related essentially with the quantity of fat. In a general way the results were slightly lower than those reported for other green table olives [17,23]. This reduced caloric intake is important from a nutritional point of view and is associated, as discussed, to the state of maturity of the olives and to the processing method itself. 

### 2.2. Fatty Acids Composition

Table 2 shows the detailed fatty acid composition (relative percentage within the lipid fraction) for “*alcaparras*” stoned table olives for the three years of study.

**Table 2:** Fatty acid composition (percentage of total fatty acids as methyl esters) of oil extracted from “*alcaparras*” stoned table olives samples from three different years of production (mean ± SD).

Fatty acid	2004	2005	2006	Average
Palmitic acid (C_16:0)_	12.49 ± 0.94 b	12.88 ± 0.95 a,b	13.66 ± 0.76 a	**13.3 ± 0.9**
Palmitoleic acid (C_16:1_)	0.84 ± 0.15 b	0.95 ± 0.21 a,b	1.05 ± 0.08 a	**1.0 ± 0.2**
Heptadecanoic acid (C_17:0_)	0.11 ± 0.07 a	0.12 ± 0.08 a	0.08 ± 0.06 a	**0.1 ± 0.1**
Heptadecenoic acid (C_17:1_)	0.14 ± 0.10 a	0.18 ± 0.13 a	0.15 ± 0.09 a	**0.2 ± 0.1**
Stearic acid (C_18:0_)	2.34 ± 0.36 a,b	2.75 ± 0.51 a	2.15 ± 0.34 b	**2.4 ± 0.5**
Oleic acid (C_18:1_)	78.43 ± 2.80 a	77.11 ± 1.37 a	77.79 ± 1.30 a	**77.4 ± 1.3**
Linoleic acid (C_18:2_)	4.11 ± 2.39 a	4.26 ± 1.17 a	3.06 ± 0.55 a	**3.7 ± 1.1**
Linolenic acid (C_18:3_)	0.66 ± 0.06 b	0.73 ± 0.07 b	0.88 ± 0.09 a	**0.8 ± 0.1**
Arachidic acid (C_20:0_)	0.23 ± 0.06 b	0.44 ± 0.06 a	0.47 ± 0.04 a	**0.5 ± 0.0**
Eicosenopic acid (C_20:1_)	0.34 ± 0.03 b	0.30 ± 0.03 c	0.39 ± 0.02 a	**0.4 ± 0.1**
Behenic acid (C_22:0_)	0.14 ± 0.02 a,b	0.13 ± 0.02 b	0.16 ± 0.03 a	**0.2 ± 0.0**
Lignoceric acid (C_24:0_)	0.08 ± 0.01 b	0.03 ± 0.01 c	0.14 ± 0.06 a	**0.1 ± 0.1**
SFA	15.43 ± 0.77 b	16.41 ± 0.78 a,b	16.46 ± 1.25 a	**16.5 ± 0.9**
MUFA	79.75 ± 2.86 a	78.56 ± 1.46 a	79.89 ± 1.27 a	**79.2 ± 1.5**
PUFA	4.77 ± 2.41 a	4.99 ± 1.21 a	3.44 ± 0.55 a	**4.2 ± 1.2**
*trans *isomers	0.05 ± 0.01 a	0.04 ± 0.01 a	0.05 ± 0.05 a	**0.04 ± 0.03**

Different letters in the same row indicate significant differences between years (*P* < 0.05).

Oleic acid is the predominant one (77.4%), ranging from 70.9% (Sample 4) to 81.8% (Sample 9). Palmitic acid was the second most abundant fatty acid, followed by linoleic acid, and stearic acid, a pattern observed in the three years, and common to most reported data [7,17,33,39]. These results are also in accordance with those regulated for olive oil [27]. The composition of fatty acids in the analyzed samples, as expected, showed a similar composition to the olive oils produced in the region [40,41].

The fatty acid analysis allowed the estimation of the different nutritional fractions (Saturated Fatty Acids – SFA; Monounsaturated Fatty Acids – MUFA, Polyunsaturated Fatty Acids – PUFA and *trans* isomers), expressed as relative percentage of total fatty acids (Table 2). MUFA were the major group, without significant differences between the three years of study. SFA represented less than 18.2% (Sample 9), with an average of 16.5%. Concerning PUFA, their amount represented less than 5.0%. The *trans* fatty acids had a very limited occurrence (less than 0.04%). In opposition to the variations in the nutritional data (Table 1), a high homogeneity was observed in the fatty acids. With a PUFA:SFA ratio near 0.3 and a (MUFA + PUFA):SFA of 5 (Table 4), the “*alcaparras*” stoned table olives’ lipid fraction is mainly characterized by a high oleic acid content, within that reported for olive oil, exhibiting, therefore, some of the nutritional benefits of the former.

### 2.3. Tocopherols Composition

The results obtained for the tocopherols content are reported in Table 3.

**Table 3:** Tocopherols content (µg/g) of oil extracted from “*alcaparras*” pitted table olives from two different years of production (mean ± SD).

Samples	α-tocopherol	β-tocopherol	γ-tocopherol	δ-tocopherol	Total
2005					
*11*	67.4 ± 1.0	1.7 ± 0.2	1.2 ± 0.1	0.3 ± 0.0	70.7 ± 1.2
*12*	68.7 ± 0.2	0.7 ± 0.0	9.7 ± 0.3	0.4 ± 0.0	79.7 ± 0.2
*13*	77.1 ± 0.3	1.2 ± 0.0	1.0 ± 0.1	0.1 ± 0.0	79.3 ± 0.4
*14*	78.5 ± 1.5	1.1 ± 0.2	3.4 ± 0.2	0.1 ± 0.0	83.1 ± 1.8
*15*	65.8 ± 0.7	1.0 ± 0.1	2.2 ± 0.2	0.1 ± 0.0	69.1 ± 0.9
*16*	52.5 ± 1.7	1.0 ± 0.1	2.3 ± 0.4	0.1 ± 0.0	55.8 ± 2.1
*17*	47.8 ± 0.3	1.0 ± 0.1	7.3 ± 0.2	0.3 ± 0.0	56.4 ± 0.5
*18*	62.5 ± 2.9	0.8 ± 0.1	0.7 ± 0.2	0.1 ± 0.0	64.2 ± 3.2
*19*	81.5 ± 3.9	1.4 ± 0.2	2.7 ± 0.4	0.1 ± 0.0	85.7 ± 4.3
*20*	57.4 ± 5.1	1.0 ± 0.1	2.5 ± 0.2	0.2 ± 0.0	60.4 ± 6.1
Average	**65.9 ± 11.0 a**	**1.0 ± 0.3 a**	**3.3 ± 2.9 a**	**0.2 ± 0.1 a**	**70.4 ± 11.0 a**
2006					
*21*	60.8 ± 3.8	7.0 ± 0.5	3.0 ± 0.1	0.3 ± 0.0	71.2 ± 4.2
*22*	45.0 ± 2.0	1.2 ± 0.1	1.0 ± 0.1	0.4 ± 0.0	47.6 ± 2.1
*23*	66.3 ± 2.7	0.6 ± 0.0	3.0 ± 0.2	0.1 ± 0.0	70.1 ± 2.8
*24*	58.1 ± 3.5	1.0 ± 0.1	3.2 ± 0.4	0.1 ± 0.0	62.5 ± 3.9
*25*	83.3 ± 4.1	0.7 ± 0.0	4.4 ± 0.2	0.3 ± 0.0	88.8 ± 4.3
*26*	87.7 ± 4.6	1.4 ± 0.1	2.3 ± 0.2	0.1 ± 0.0	91.6 ± 4.9
*27*	53.9 ± 0.3	1.4 ± 0.0	0.9 ± 0.1	0.3 ± 0.2	56.4 ± 0.1
*28*	30.7 ± 0.6	1.2 ± 0.0	1.5 ± 0.0	0.2 ± 0.1	33.7 ± 0.5
*29*	55.7 ± 4.4	0.7 ± 0.0	3.9 ± 0.3	0.5 ± 0.0	60.8 ± 4.6
*30*	106.3 ± 4.2	1.6 ± 0.2	1.1 ± 0.1	0.6 ± 0.0	109.5 ± 4.4
Average	**64.8 ± 21.6 a**	**1.7 ± 1.8 a**	**2.4 ± 1.2 a**	**0.3 ± 0.2 a**	**69.2 ± 21.9 a**

Different letters in the same row indicates significant differences between years (*P* < 0.05).

In the first year of sample collection, 2004, the tocopherols analysis were not performed immediately, and the measures taken to conserve the samples were ineffective, resulting in lower amounts when compared with the following years, and therefore they not taken into account in our analysis of the data. In 2005 and 2006 the analysis were performed within a shorter period. All samples presented the four tocopherol isomers α, β, γ and δ, although this last in vestigial amounts.

The more representative was α-tocopherol, with an average content of 69.8 µg/g of extracted lipids, with no significant differences between the two sampling years, and equivalent to 1.20 mg/100 g of fresh weight. These values are generally lower than those reported for other table olives, with 2 mg/100 g on average reported by Montaño *et al*. [5] and 4 to 8 mg/100 g by Sakouhi *et al*. [7]. Taking into account the preparation method of “*alcaparras*” stoned table olives with a constant exposure of the pulp with water during processing and later to brine, it can promote exposure of the inner fat to oxidation, with an increased reduction of vitamin E. Still, the values obtained demonstrate that this kind of table olives contribute to the nutritional requirements of vitamin E, with a serving size (20 g) contributing approximately with 10% of the recommended daily intake of tocopherols [26]. The high phenolic content of these traditional stoned table olives, derived from the maturity as picking and soft processing conditions, protects the products against autoxidation and microbial development [21] while increasing the functional value of this product.

### 2.4. Statistical Analysis

**Figure 1:**
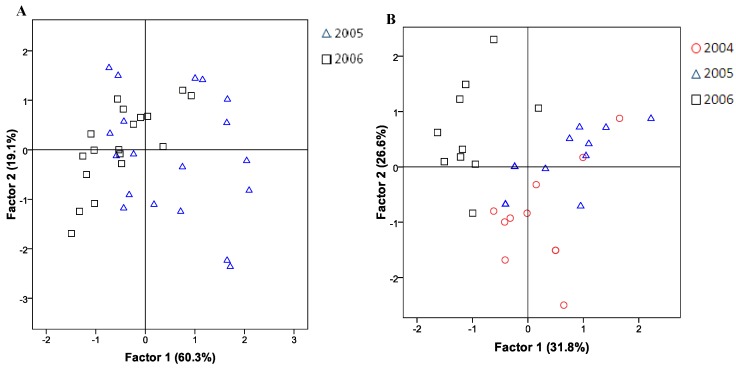
Principal components analysis using chemical composition data (A – 2005 and 2006) and fatty acids data (B – 2004 to 2006) of commercial stoned table olives “*alcaparras”*. The PCA factors explain 79.4% and 58.4% (A and B respectively) of the total variance.

Due to the high variability observed between the samples, and the significance observed for some parameters, a principal component analysis (PCA) was applied to the chemical composition and nutritional values obtained for the samples analyzed in 2005 and 2006 Figure 1A. Another PCA was applied to the fatty acids profile of “*alcaparras*” stoned table olives from the three years of production Figure 1B.

Figure 1A shows the two-dimensional representation of the two principal components factor scores. The results extracted from the PCA shown that 79.4% of the total variance of the data obtained could be explained using only two principal components, with a partial separation of samples into two groups.

The first principal component factor allowed separating the samples from 2005 from those of 2006. The samples from 2005 are mainly located in the positive region, reporting higher average values of crude protein, fat and consequently higher energy content, a fact that is probably associated with a higher proportion in the final product of olive cultivars with high fat contents, which characterized the samples from 2005. Samples from 2006 are represented mainly in the negative region of the first principal component, due to their higher water content. There is a strong possibility that the technological treatment might have been longer in the samples produced in 2006 than those from 2005, which explain the high moisture in the samples.

The higher homogeneity in the fatty acid composition is also visible in the PCA applied to the fatty acid profiles. Figure 1B shows the two-dimensional representation of the two principal components factor scores obtained, allowing explaining 58.4% of the total variance. From the information obtained from the PCA, although not allowing a clear separation of the samples associated to the three years, a tendency was observed. Thus, the samples from 2004 are mainly located in the negative region of the second principal component once that this samples reported higher average values of MUFA and oleic acid (C_18:1_). In the positive region of the first principal component are located the samples from the year 2005 due to their higher PUFA content. The samples from the year of 2006 are mainly located in the negative region of the first principal component due to the higher SFA and eicosenoic acid (C_20:1_). The fatty acids profile was mainly influenced by the olive cultivars that composed the final product, and the variation accounted derived mainly from that fact.

**Figure 2:**
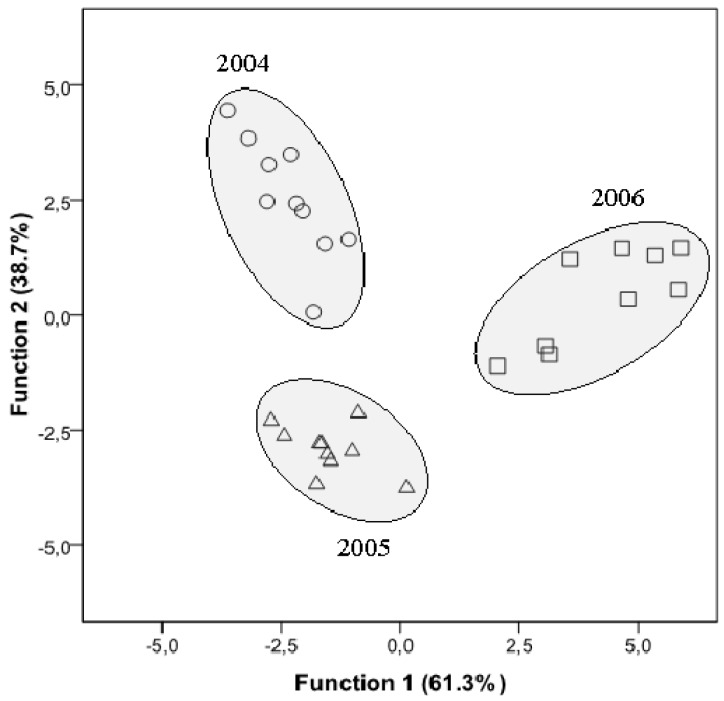
Linear discriminant analysis of the fatty acids profile from commercial “*alcaparras*” stoned table olives from three different years, represented in a plane composed by the two main discriminant functions. The functions explain 100% of the total variance.

Using a linear discriminant analysis (LDA) on the same fatty acid data used in the PCA, it was possible to discriminate the samples from the three years (Figure 2). Only two significant discriminant functions were used once they could explain 100% of the observed variance. 

Based only in three variables (C_24:0_, C_20:0_ and C_18:0_) the model showed a very satisfactory classification performance allowing to correctly classifying all the samples for the original groups as well as for the cross-validation procedure (sensibilities and specificities of 100%).

Like in the others parameters studied, the tocopherols content was also subjected to a PCA. In this case the variance observed was reduced among the years in study and therefore with the results obtained from the PCA was difficult to observe which variables characterize each sampling year.

## 3. Experimental

### 3.1. Samples

A total of 30 “*alcaparras*” stoned table olives samples were studied during three years (2004–2006). In each year, between October and December, ten samples were purchased from local markets in different municipalities of Trás-os-Montes region: Bragança, Mirandela, Carrazeda de Ansiães and Macedo de Cavaleiros. Each sample, of approximately 1 kg weight, was drained, conditioned in plastic bags, and frozen to −20 °C until analysis. All determinations were carried out at least in duplicate. 

### 3.2. Samples Preparation

For proximate chemical composition (moisture, protein, fat, ash), “*alcaparras*” stoned table olives samples were chopped in a 643 MX mill (Moulinex, Spain). For fatty acid and tocopherol profiles the lipids were extracted after being further triturated in an Ultra-Turrax (T-25 Janke & Kunkel IKA, Staufen, Germany). The olive paste was homogenized and warmed up in a water bath (35 °C), during 30 minutes, and the oil was extracted two times by centrifugation (5,000 r.p.m./30 min) (K240R, Centurion Scientific). The oil was decanted, filtered in the presence of anhydrous sodium sulphate, and stored at 4 °C in aluminium foil wrapped falcon tubes, until analysis.

### 3.3. Chemical Analysis

#### 3.3.1. Pulp Analysis

Moisture, total fat, ash and protein contents were analyzed in duplicate. Moisture was determined at 100 ± 2 °C (~5 g test sample) by AOAC 925.40 [24] method. Total fat content was determined in a Soxhlet apparatus according to AOAC 948.22 [25] method using petroleum ether as solvent with a minimum extraction time of 24 h. Crude protein content was estimated by the Kjeldahl method and ash content was determined by incineration at 550 ± 15 °C until consistent weight [25]. Carbohydrate content was estimated by difference of the other components. Energy was expressed as kilocalories using regulated conversion factors [26] as follows:

Energetic value (kcal) = [(g protein + g carbohydrates) × 4] + g fat × 9

### 3.4. Oil Analysis

#### 3.4.1. Fatty Acid Composition

Fatty acids were evaluated as their methyl esters after alkaline transesterification with methanolic potassium hydroxide solution [27] and extraction with *n-*heptane. The fatty acid profile was determined by GC-FID (Chrompack CP 9001, Middelburg The Netherlands) equipped with a split-splitless injector, and a 50 m × 0.25 mm i.d. CP-Sil 88 column (manufactured by Chrompack and available from Varian Inc.). Helium was used as carrier gas at an internal pressure of 120 kPa. The results are expressed in relative percentage of each fatty acid, calculated by internal normalization of the chromatographic peak area. A control sample (olive oil 47118, Supelco) and a fatty acids methyl esters standard mixture (Supelco 37 FAME Mix) were used for identification and calibration purposes (Sigma, Spain).

#### 3.4.2. Tocopherol Composition

Tocopherols were evaluated following the ISO 9936 international standard [28], with some modifications as implemented by Amaral *et al*. [29]. A sample of extracted oil (50 mg) was blended with an appropriate amount of internal standard (tocol, Matreya, Inc.) in a volume of *n*-hexane (1.5 mL), homogenized by stirring, centrifuged at 13,000 g and analyzed by HPLC. The liquid chromatograph consisted of a Jasco integrated system (Japan) equipped with an AS-950 automated injector, a PU-980 pump, an MD-910 multiwavelength diode array detector and an FP-920 fluorescence detector (λ_ex_ = 290 nm and λ_em_ = 330 nm), connected in series. The chromatographic separation was achieved on a Supelcosil ^TM^ LC-SI (3 μm) 75 × 3.0 mm (Supelco, Bellefonte, PA, USA), operating at constant room temperature (21 °C). A 98:2 mixture of *n*-hexane and 1,4-dioxane was used as eluent, at 0.7 mL/min. Data were processed by the Borwin PDA Controller Software (JMBS, France). Tocopherols (α, β, γ, and δ) were identified by chromatographic comparisons with authentic standards, by co-elution and by their UV spectra. Quantification was based on the internal standard method, using the fluorescence signal response.

### 3.5. Statistical Analysis

One-way analysis of variance (one-way ANOVA), principal components analysis (PCA), and the linear discriminant analysis (LDA) were performed using SPSS software, version 17.0 (SPSS, Inc., Chicago, IL, USA). All data were analysed by one-way ANOVA followed by Tukey’s HSD Test with α = 0.05. Principal components analysis (PCA) was applied for reducing the number of variables (17 variables corresponding to the fatty acids profile of the three years and 6 variables corresponding to the chemical composition from 2005 and 2006) to a smaller number of new derived variables (principal component or factors) that adequately summarize the original information, *i.e.*, the chemical composition from three different years, 2004, 2005 and 2006. Moreover, it allowed recognizing patterns in the data by plotting them in a multidimensional space, using the new derived variables as dimensions (factor scores).

A linear discriminant analysis (LDA) was used as a supervised learning technique to classify the samples from the three different years according to their fatty acids profile. A stepwise technique, using the Wilk’s lambda method with the usual probabilities of F (3.84 to enter and 2.71 to remove), was applied for variable selection. This procedure uses a combination of forward selection and backward elimination procedures, where before selecting a new variable to be included, it is verified whether all variables previously selected remain significant [30,31]. With this approach, it is possible to identify the significant variables among the fatty acids profile obtained for each year. To verify which canonical discriminant functions were significant, the Wilks’ Lambda test was applied. To avoid overoptimistic data modulation, a leaving-one-out cross-validation procedure was carried out to assess the model performance. Moreover, the sensibility and specificity of the discriminant model were computed from the number of individuals correctly predicted as belonging to an assigned group [30,32]. Sensibility was calculated by dividing the number of samples of a specific group correctly classified by the total number of samples belonging to that specific group. Specificity was calculated by dividing the number of samples of a specific group classified as belonging to that group by the total number of samples of any group classified as belonging to that specific group.

## 4. Conclusions

Based in the data obtained in the present study and in the bibliography, mean nutritional characteristics and chemical composition of “*alcaparras*” stoned table olives are compiled in Table 4 for 100 g fresh weight and for an estimated serving size of 20 pieces, equivalent to 10 olives.

**Table 4:** Nutritional characteristics of “*alcaparras*” stoned table olives.

	100 g	Serving size ^a^
Energy (kcal)	156	31
Energy (kJ)	652	130
Edible part	100%	
Moisture (g)	72.5	14.5
Proteins (g)	1.1	0.2
Minerals (g)	3.4	0.7
Carbohydrates (g) ^b^	4.6	0.9
Fibre (g)	2.7 ^c^	0.5
Fat (g)	14.6	2.5
Saturated fatty acids	2.1	0.4
Monounsaturated fatty acids	10.0	2.5
Polyunsaturated fatty acids	0.5	0.1
*Trans* fatty acids	tr	tr
PUFA:SFA	0.3	
(MUFA + PUFA):SFA	5.0	
Vitamin E (mg)	1.2	0.3
Polyphenols (g) ^d^	1.3	0.3

Data are expressed as mean of the 30 commercial samples analyzed. ^a^ equivalent to 20 pieces (10 olives – 20 g); ^b^ estimated; ^c^ based on reported data: [36]; ^d^ based on reported data: [20].

Commercial “*alcaparras*” stoned table olives can be regarded as important sources of MUFA, like olive oil, recognized as a preventive factor in diseases in which free radicals are implicated, complemented by the vitamin E amounts, with both antioxidant and vitamin action. The relative low caloric intake in comparison with other table olives is also important from a nutritional point of view, while satisfying the increasing consumers demand for traditional and functional products.

Previous works have demonstrated that “*alcaparras*” stoned table olives are also a source of a appreciable amount of other molecules with high value such phenolic compounds [21,42] and volatiles [43]. 

With the obtained data we understand that the role of the different olive cultivars and their ripening stage in “*alcaparras*” stoned table olives processing need to be studied and clarified. The standardization of the mineral amount should be established by the producers, once that its content is mainly derived by the addition of NaCl, used as a preservation factor. Chemometrics demonstrate that when this kind of studies are developed it is imperative to work with a significant amount of samples from different years of production, once that in each year different aspects influence the chemical composition of “*alcaparras*” stoned table olives. 
